# Comparison of three diagnostic methods (microscopy, RDT, and PCR) for the detection of malaria parasites in representative samples from Equatorial Guinea

**DOI:** 10.1186/s12936-018-2481-4

**Published:** 2018-09-17

**Authors:** Pedro Berzosa, Aida de Lucio, María Romay-Barja, Zaida Herrador, Vicenta González, Luz García, Amalia Fernández-Martínez, Maria Santana-Morales, Policarpo Ncogo, Basilio Valladares, Matilde Riloha, Agustín Benito

**Affiliations:** 1 Malaria Laboratory, National Centre of Tropical Medicine, Institute of Health Carlos III, C/Monforte de Lemos 5, 28029 Madrid, Spain; 2 Network Collaborative Research in Tropical Diseases, RICET, Madrid, Spain; 3 Reference Centre for Control of Endemic Diseases (CRCE), Malabo, Equatorial Guinea; 4 Ministry of Health and Social Welfare of Equatorial Guinea, Malabo, Equatorial Guinea; 5 Instituto Universitario de Enfermedades Tropicales y Salud Pública de Canarias, Universidad de la Laguna, Tenerife, Spain

**Keywords:** Malaria, Diagnosis, Microscopy, RDTs, SnM-PCR

## Abstract

**Background:**

Malaria in Equatorial Guinea remains a major public health problem. The country is a holo-endemic area with a year-round transmission pattern. In 2016, the prevalence of malaria was 12.09% and malaria caused 15% of deaths among children under 5 years. In the Continental Region, 95.2% of malaria infections were *Plasmodium falciparum*, 9.5% *Plasmodium vivax*, and eight cases mixed infection in 2011. The main strategy for malaria control is quick and accurate diagnosis followed by effective treatment. Early and accurate diagnosis of malaria is essential for both effective disease management and malaria surveillance. The quality of malaria diagnosis is important in all settings, as misdiagnosis can result in significant morbidity and mortality. Microscopy and RDTs are the primary choices for diagnosing malaria in the field. However, false-negative results may delay treatment and increase the number of persons capable of infecting mosquitoes in the community. The present study analysed the performance of microscopy and RDTs, the two main techniques used in Equatorial Guinea for the diagnosis of malaria, compared to semi-nested multiplex PCR (SnM-PCR).

**Results:**

A total of 1724 samples tested by microscopy, RDT, and SnM-PCR were analysed. Among the negative samples detected by microscopy, 335 (19.4%) were false negatives. On the other hand, the negative samples detected by RDT, 128 (13.3%) were false negatives based on PCR. This finding is important, especially since it is a group of patients who did not receive antimalarial treatment.

**Conclusions:**

Owing to the high number of false negatives in microscopy, it is necessary to reinforce training in microscopy, the “Gold Standard” in endemic areas. A network of reference centres could potentially support ongoing diagnostic and control efforts made by malaria control programmes in the long term, as the National Centre of Tropical Medicine currently supports the National Programme against Malaria of Equatorial Guinea to perform all of the molecular studies necessary for disease control. Taking into account the results obtained with the RDTs, an exhaustive study of the deletion of the *hrp2* gene must be done in EG to help choose the correct RDT for this area.

## Background

Equatorial Guinea (EG) in Central West Africa is divided into two regions, the Insular Region (Bioko, Annobon) and the Continental Region (Rio Muni). Malaria remains a major public health problem in the country, which is a holo-endemic area with a year-round transmission pattern [1]. According to the 2017 World Health Organization (WHO) Malaria Report, the prevalence of malaria in the country was 12.09% in 2016 [2]. This parasitic disease accounts for 15% of deaths among children under 5 years of age. In the Continental Region, 95.2% of malaria infections were *Plasmodium falciparum*, 9.5% *Plasmodium vivax*, and eight cases mixed infection in 2011 [3].

The main strategy for malaria control is quick and accurate diagnosis followed by effective treatment [4]. The early and accurate diagnosis of malaria is essential for both effective disease management and malaria surveillance. The quality of malaria diagnosis is important in all settings, as misdiagnosis can result in significant morbidity and mortality. Since 2010, the WHO has recommended that all patients with suspected malaria should have their diagnosis confirmed by microscopy or a rapid diagnostic test (RDT) before treatment [5]. Microscopy and RDTs are the primary choices for diagnosing malaria in the field. However, false-negative results may delay treatment and increase the number of persons capable of infecting mosquitoes in the community.

Microscopy is still considered the “gold standard” for malaria diagnosis in endemic countries. This method has a sensitivity of 50–500 parasites/μl [6], is inexpensive, and allows the identification of species and parasite density [7, 8]. It is necessary to observe many fields to detect infection, which implies at least two expert microscopists. However, the quality of microscopy-based diagnosis is frequently inadequate [9]. In many malaria-endemic regions, microscopic diagnosis has certain limitations, including a shortage of skilled microscopists, inadequate quality control, and the possibility of misdiagnosis due to low parasitaemia or mixed infections [10–12]. In EG, this diagnostic method is unavailable in some rural health facilities, and predictive diagnosis is still widely used [13, 14]. Moreover, sometimes it is difficult to determine the species of *Plasmodium* by microscopy; consequently, some species are not reported in the country, such as *Plasmodium ovale*, which has a morphology similar to *P. vivax*. Even if this does not affect treatment, because the patient will receive the same treatment for both species, it has important implications in malaria epidemiology and mapping [15].

Microscopy has low sensitivity when performed by poorly trained personnel in endemic areas, especially in primary and secondary healthcare facilities. This may result the over- or under-diagnosis of malaria, with excessive use of anti-malarial drugs or negligent treatment, which invariably contributes to malaria morbidity and the development of resistance [16]. Therefore, in the absence of well-prepared technicians for microscopic diagnosis in many areas of sub-Saharan Africa, the WHO recommends RDTs as a good alternative method for malaria diagnosis [16, 17]. In remote parts of sub-Saharan Africa, RDTs have become the primary tool for the parasitological diagnosis or confirmation of malaria [18].

The most widely used RDTs for malaria are based on the detection of parasite histidine-rich protein II (HRP2), in addition to *Plasmodium* lactate dehydrogenase (pLDH) or p-aldolase detection, molecules produced by the parasite during the erythrocytic cycle [16]. RDTs have a sensitivity of ~ 100 parasites/μl [6]. The major constraint of RDTs are false positives, because HRP2 persists in the blood for several days after infection clearance [19], and false negatives due to gene deletions, which were recently reported for HRP2 in field isolates from Eritrea [20]. In addition, these tests are thought to not be very reliable for non-Pf infections [21].

Regarding the molecular detection of malaria, the WHO recommends that nucleic acid amplification tests be considered only for epidemiological research and survey mapping sub-microscopic infections. Implementation of the molecular techniques as diagnostic methods in sub-Saharan Africa is complicated due to the equipment required, reagent maintenance, and the qualified personnel required. Polymerase chain reaction (PCR) is the most sensitive method available, detecting parasitemia as low as 2–5 parasites/μl. However, it is not appropriate for use in the field, as it is an expensive and complex method.

The objective of the present study was to analyse the performance of microscopy and RDTs, the two main techniques used in EG for the diagnosis of malaria, compared to semi-nested multiplex PCR (SnM-PCR).

## Methods

### Study area

**Fig. 1:**
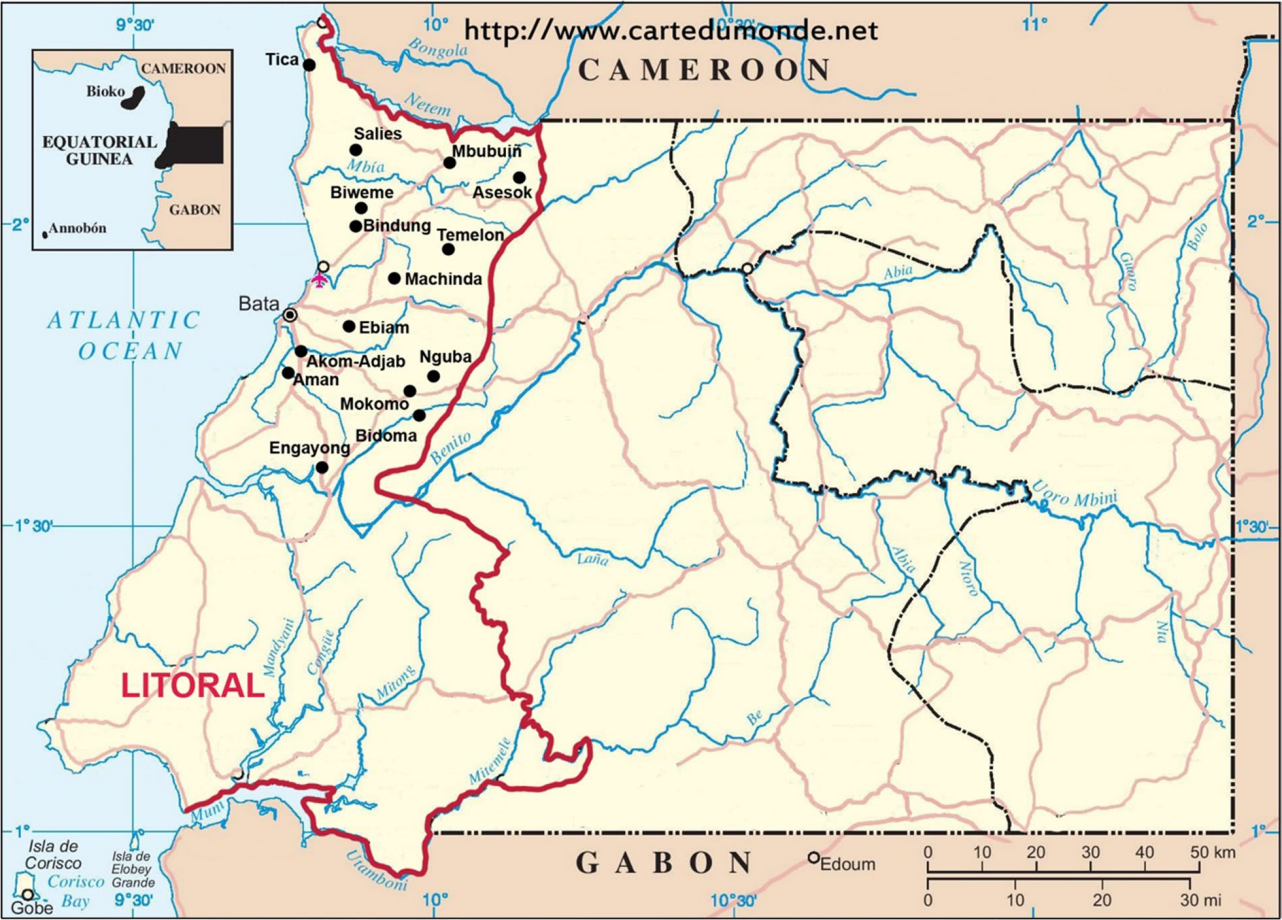
Geographic map of the Continental Region. The red line marks the limits of the Litoral Province, where the study was carried out, whose capital is Bata. The different villages where the samples were collected are indicated in the map [Source: http://www.cartedumonde.net with modifications (National Center of Tropical Medicine-ISCIII; also, this Figure appears in Berzosa et al. Malar J. 2017;16:28)]

The survey was carried out in the district of Bata in Litoral Province of the Continental Region of EG, located between Cameroon and Gabon (Fig. 1). The region has a tropical climate with two dry seasons (December to March and June to September) alternating with two rainy seasons (March to June and September to December). The mean daily maximum and minimum temperatures are 29–32 and 19–22 °C, respectively.

### Study population

A cross-sectional survey was carried out June–August 2013 in Bata as part of a project called “PREVAMAL”, which aimed to provide baseline data on malaria prevalence, molecular characterization of *Plasmodium* and malaria vectors in the area, and information on the knowledge, practices, and attitudes among the targeted population [22]. Sampling was carried out using a multistage, stratified cluster strategy, assuming an expected malaria prevalence of 50%. Rural villages and urban neighbourhoods were randomly selected with probability proportional to the size to improve accuracy in sample design. A total of 1741 individuals (1043 and 698 people living in urban and rural settings, respectively) were finally recruited [22]. Other methodological aspects have been described elsewhere [23, 24].

Blood samples were taken from the finger for the diagnosis of malaria using malaria RDTs and microscopy. The blood was spotted on Whatman 903™ paper (GE Healthcare Bio-Sciences Corp.) for further molecular studies. The blood on the filter paper was air dried, stored in double zip-lock plastic bags with silica gel at 4 °C, and subsequently transported to the National Centre for Tropical Medicine, Institute of Health Carlos III, Madrid (Spain) for diagnostic confirmation by PCR.

For the purpose of this study, only the 1724 samples tested by the three diagnostic methods (RDT, Giemsa microscopy, and SnM-PCR) were evaluated. PCR was considered the gold standard.

### Microscopy

Thin and thick slides were made in the participants’ homes, where the blood samples were also taken on Whatman paper. Slides of the peripheral blood specimens were made immediately after collection on a clean, grease-free microscope slide and allowed to air dry. The films were stained with 10% Giemsa solution (Appichem, Panreac ITW Companies) for 10 min and examined by microscopists from the Malaria National Programme of the Ministry of Health and Social Welfare of Equatorial Guinea, health centers, and Bata Hospital. The slides were allowed to air dry and subsequently examined by light microscopy using an oil immersion objective lens. A slide was declared negative only after observing 100 microscopic fields without finding parasites. For each specimen, the thick films were examined first for the detection of malaria parasites. The thin films of each specimen in which malaria parasites were identified in the thick film were subsequently examined for speciation. The slides were examined by two microscopists; each microscopist examined each specimen independently and the results were recorded as positive when both microscopists recorded a positive result and the same species. When there was a discrepancy, a third microscopist assessed the slide. The microscopists observed the slides without knowing the previous diagnosis obtained with the RDTs.

### Rapid diagnostic test

The NADAL^®^ Malaria 4 species test (Test cassette) (Nal von Minden, Moers, Germany) was used as the RDT in situ. The test enables differential diagnosis between *P. falciparum*, *Plasmodium malariae*, *P. vivax*, and *P. ovale* in human whole blood samples. The test is based on the detection of HRP2 specific for *P. falciparum* and pLDH specific for *Plasmodium* sp. The test has a sensitivity of 99.7% for *P. falciparum* and 95.5% for non-falciparum with the microscopic detail of a large droplet, and a specificity of 99.5%. The test detects the HRP2 and pLDH proteins; the cut-off level was 1–50 parasites/μl of blood for HRP2 and 51–100 parasites/μl of blood for pLDH. To perform the malaria test, 5 μl of whole blood was collected with the provided capillary pipette and transferred to the sample well. Four drops of the assay diluent were added to the diluent well according to the manufacturer’s protocol. The results were read after 15 min. Participants with positive RDTs were immediately offered treatment according to national guidelines [23].

### DNA extraction and molecular analysis

DNA was extracted from the filter paper samples using commercial kits (Speedtools tissue DNA Extraction Kit, Biotools, Spain). SnM-PCR was performed as described previously [24, 25]. The method is based on features of the small subunit nuclear ribosomal RNA gene (ssrDNA), a multicopy gene possessing both highly conserved domains and domains characteristic of each of the four human malaria parasites. The first reaction in SnM-PCR includes a universal reverse primer with two forward primers specific for *Plasmodium* and mammals, respectively. The mammalian-specific primer was included as a positive control to distinguish uninfected cases from simple PCR failures. The second PCR reaction includes a *Plasmodium*-specific forward primer plus species-specific reverse primers for *P. falciparum, P. vivax, P. malariae* and *P. ovale*. The technique is more sensitive and specific than the standard microscopic examination [26, 27]. The SnM-PCR used in this study for the diagnosis of malaria has a sensitivity of 0.0001 parasites/μl [26]. Diagnostic PCR was performed for all samples positive for malaria by the other methods and, in line with quality assurance programmes, the PCR was performed for 10% of all negative samples. If any negative samples were positive by PCR, 10% were taken again, and so on until no more positive samples appeared. Finally, SnM-PCR was performed with the 1724 samples.

### Statistical analysis

Frequencies with 95% confidence intervals (CIs) were used for categorical variables. Associations were assessed by the Chi square test or Fisher’s exact test. The level of significance was set at P ≤ 0.05. Statistical analyses were performed using the software package SPSSv.15.0. Sensitivity and specificity calculations for microscopy and RDT were performed using Epidat 3.1 software and were calculated using SnM-PCR as the reference technique, “Gold Standard”. Stratified sensitivity and specificity analysis by age and place of origin (rural and urban) was also performed.

### Ethics

This study was approved by the Minister of Health and Social Welfare of Equatorial Guinea (MINSABS) and the Ethics Committee of the Spanish National Health Institute, Carlos III (CEI PI 22_2013-v3). Written informed consent was obtained from all participants.

## Results

A total of 1724 samples tested by microscopy, RDT, and SnM-PCR were analysed. The results are summarized in Table 1. In two positive samples it was not possible to determine the species by microscopy and in 71 samples it was not possible to determine if were positive or negative because the staining was not good or the slide were deteriorated. Significant differences were found among the positive and negative samples and in the different species detected when comparing the three diagnostic methods (Table 1).

**Table 1:** Diagnostic results with each method

	RDT			Microscopy			SnM-PCR			*P-value*
	N	%	95% CI	N	%	95% CI	N	%	95% CI	
Negative samples	963	56	55.3–58.2	1069	62	59.7–64.3	937	54	52–56.7	*< 0.001*
Positive samples	761	44	41.8–46.5	655	38	35.7–40.3	787	46	43.3–48	*< 0.001*
*P. falciparum*	527	69.2	65.9–72.4	571	87.2	84.4–89.5	763	97	95.5–97.9	*< 0.001*
*P. vivax*	–	–	–	–	–	–	1	0.1	0–0.7	–
*P. ovale*				2	0.3	0.1–1.1	5	0.6	0.3–1.5	0.457
*P. malariae*	–	–	–	9	1.4	0.7–2.6	3	0.4	0.1–1.1	*0.002*
Mixed infection	212	27.8	24.8–31.1	–	–	–	15	1.9	1.2–3.1	*< 0.001*
UK	22	2.9	1.9–4.3	2	0.3	0.1–1.1	–	–	–	*< 0.001*
WD	–	–	–	71	10.8	8.7–13.5	–	–	–	**–**

Results of the diagnosis by RDT, microscopy, and SnM-PCR, N = 1724 samples in all the cases. Appears indicated the total number of samples detected as negative and positive, within the latter the species of *Plasmodium* or mixed infections detected. UK: positive but unknown species; WD: without diagnosis. The percentage of every species was calculated in relation to the total positive samples in each case

Italic values indicate significance of *P-value* (≤ 005)

**Fig. 2:**
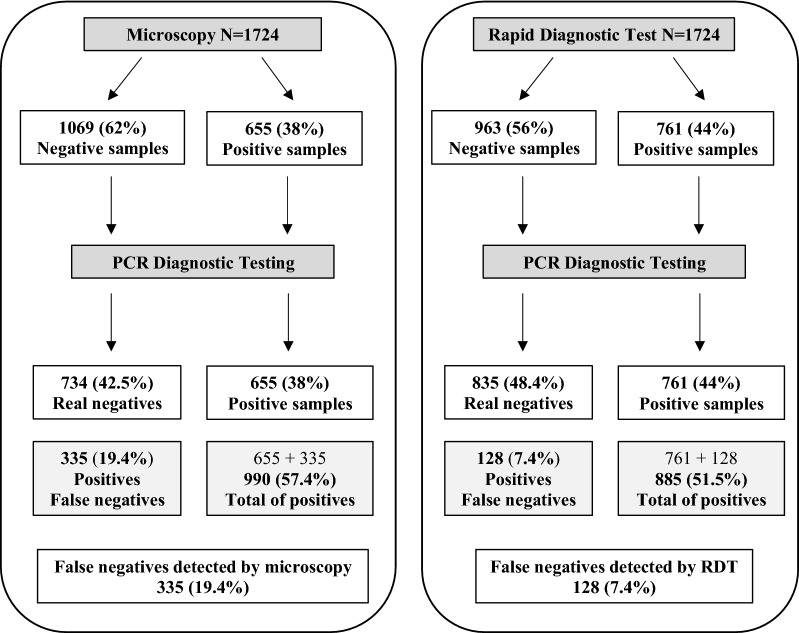
Flow diagram of diagnostic testing. Appears the processing that has been done to the 1724 samples, both microscopy and RDT. Finally, after the molecular correction by SnM-PCR, the percentage of false negatives detected in each case is indicated (19.4% in microscopy and 7.4% in RDT)

Although nine samples were diagnosed as *P. malariae* by microscopy, only one was confirmed as *P. malariae* by PCR; one was negative and the rest were *P. falciparum*. However, three samples identified as *P. malariae* by PCR were not identified as *P. malariae* by RDT or microscopy. In addition, of the 212 mixed infections detected by RDT, only 15 were mixed infections on PCR. Samples with concordant diagnoses between the methods are shown in Table 2, using PCR as the reference. Significant differences were found among positive and negative samples, and among *P. falciparum* diagnoses. The results of PCR of negative samples detected by microscopy (n = 1069) and RDT (n = 963) as a diagnostic quality control are shown in Table 3. Among the negative samples detected by RDT, 128 (13.3%) were false negatives based on PCR (Table 3). Among the negative samples detected by microscopy, 335 (19.4%) were false negatives. Figure 2 explains the processing performed for the 1724 samples.

**Table 2:** Samples that coincide in diagnosis result with SnM-PCR diagnosis

	Concordant samples PCR vs. microscopy N = 1149		Concordant samples PCR vs. RDT N = 1494		*P*-*value*	Concordant samples All techniques N = 1039		
	N	%	N	%		N	%	95% CI
Total negatives	734	64	835	56	*< 0.001*	659	63	60.5–66.3
Total positives	415	36	659	44	*< 0.001*	380	37	33.7–39.5
*P. falciparum*	393	95	428	65	*< 0.001*	209	55	50–59.9
*P. malariae*	1	0.3	–	–	0.2	–	–	–
Mixed infection	–	–	3	0.5	0.16	–	–	–

In the table appear the samples that coincide in the same diagnosis with that obtained by SnM-PCR. It compares SnM-PCR with microcopy and SnM-PCR with RDT. No samples lacked a diagnosis or were positive without a determination of species. No sample matched *P. ovale* or *P. vivax. Mic* microscopy

Italic values indicate significance of *P-value* (≤ 005)

**Table 3:** Analysis of negative samples by SnM-PCR

	RDT (n = 963)			Microscopy (n = 1069)			*P*-*value*
	N	%	95% CI	N	%	95% CI	
*P. falciparum*	122	95.3	90.2–97.8	326	97.3	95–98.6	0.276
*P. malariae*	1	0.8	0.1–4.3	1	0.3	0.1–1.7	0.47
*P. ovale*	3	2.3	0.8–6.7	2	0.6	0.2–2.2	0.10
*P. vivax*	1	0.8	0.1–4.3	1	0.3	0.1–1.7	0.47
Mix	1	0.8	0.1–4.3	5	1.5	0.6–3.4	0.47
Total positives	128	13.3	11.3–15.6	335	31.3	28.6–34.2	*< 0.001*

Negative samples obtained by RDT (963) and microscopy (1069) that were analyzed by SnM-PCR. The species and mixed infections detected are showed in the table. Samples that finally appear as positive were considered false negatives, 128 in RDT and 335 in microscopy. The frequency of false negatives in relation to the total samples analyzed (1724) was 7.4% in RDT and 19.4% in microscopy

Italic values indicate significance of *P-value* (≤ 005)

According to SnM-PCR results, they were false positives in RDT and microscopy (102 positive samples by RDTs and 203 by microscopy).

**Fig. 3:**
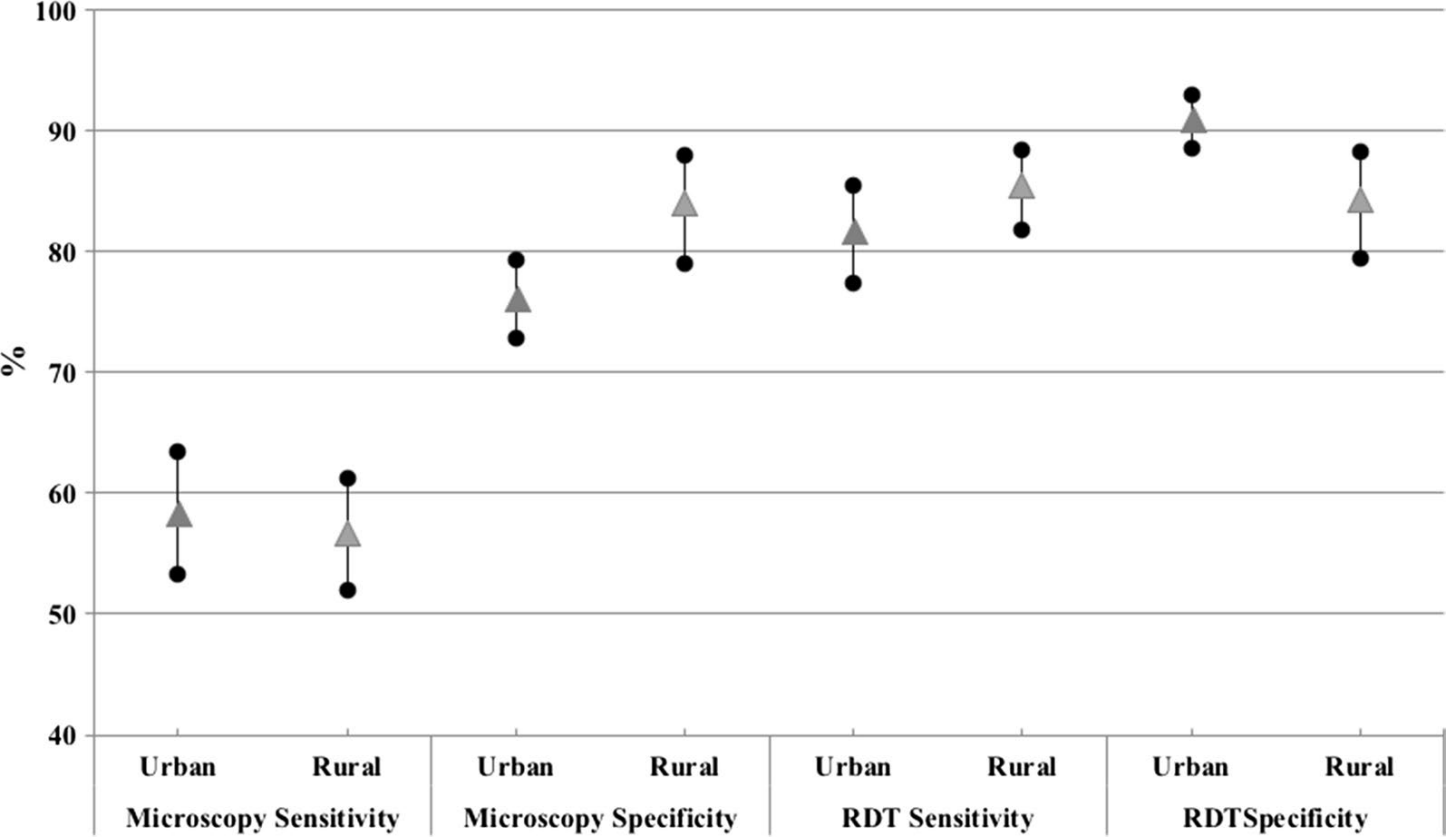
Graph of the sensitivity and specificity of microscopy and RDTs according to study area. The highlight of the graph is how the sensitivity of RDTs is higher in rural areas, while the specificity is lower

Compared to microscopy, RDTs had higher sensitivity (83.74; 95% CI 81.09–86.38) (Table 4). RDTs specificity was also higher than microscopy specificity (89.11; 95% CI 87.07–91.16 vs. 81.28; 95% CI 78.69–83.88, respectively). Regarding the specificity and sensitivity of both techniques to *P. falciparum,* RDT showed a higher sensitivity (77.8; 95% CI 74.3–81.3) and specificity (90.6; 95% CI 88.8–92.5) than microscopy (sensitivity 54.7; 95% CI 51.1–58.4 and specificity 81.5; 95% CI 79–84.1) (Table 4). When the sensitivity and specificity analysis were performed by area of residence, it was observed that the sensitivity of microscopy was similar in rural and urban areas (56.6 and 58.43, respectively), while the specificity was higher in rural areas (83.9 in rural vs. 76.2 in urban areas). Regarding the RDTs, in rural areas this test showed greater sensitivity (85.4 vs. 81.7) and lower specificity (84.3 vs. 91) than in urban settings (Table 5, Fig. 3).

**Table 4:** Sensitivity and specificity of microscopy and RDTs

	PCR/microscopy		PCR/RDT	
	Value	95% CI	Value	95% CI
Diagnosis of malaria				
Sensitivity	55.3	51.71–58.96	83.74	81.09–86.38
Specificity	81.28	78.69–83.88	89.11	87.07–91.16
Detection of *P. falciparum*				
Sensitivity	54.7	51.1–58.4	77.8	74.3–81.3
Specificity	81.5	79.0–84.1	90.6	88.8–92.5

The table shows first the sensitivity and specificity of microscopy and RDTs, taking into account the general diagnosis of malaria and without considering the different species. The second part of the table  shows the specificity and sensitivity in the detection of *P. falciparum*, considering that the RDTs permits only the diagnosis of this species with accuracy

**Table 5:** Sensitivity and specificity between settings

	PCR/microscopy		PCR/RDT	
	Value	95% CI	Value	95% CI
Urban n = 1032				
Sensitivity	58.43	(53.24–63.43)	81.74	(77.4–85.41)
Specificity	76.18	(72.83–79.24)	90.98	(88.58–92.91)
Rural n = 692				
Sensitivity	56.61	(51.9–61.21)	85.38	(81.74–88.41)
Specificity	83.91	(78.96–87.87)	84.29	(79.38–88.2)

The table shows the sensitivity and specificity of both techniques (microscopy and RDTs) in the two different settings, urban and rural. The table shows the sensitivity and specificity of both techniques (microscopy and RDTs) in the two different settings, urban and rural

**Fig. 4:**
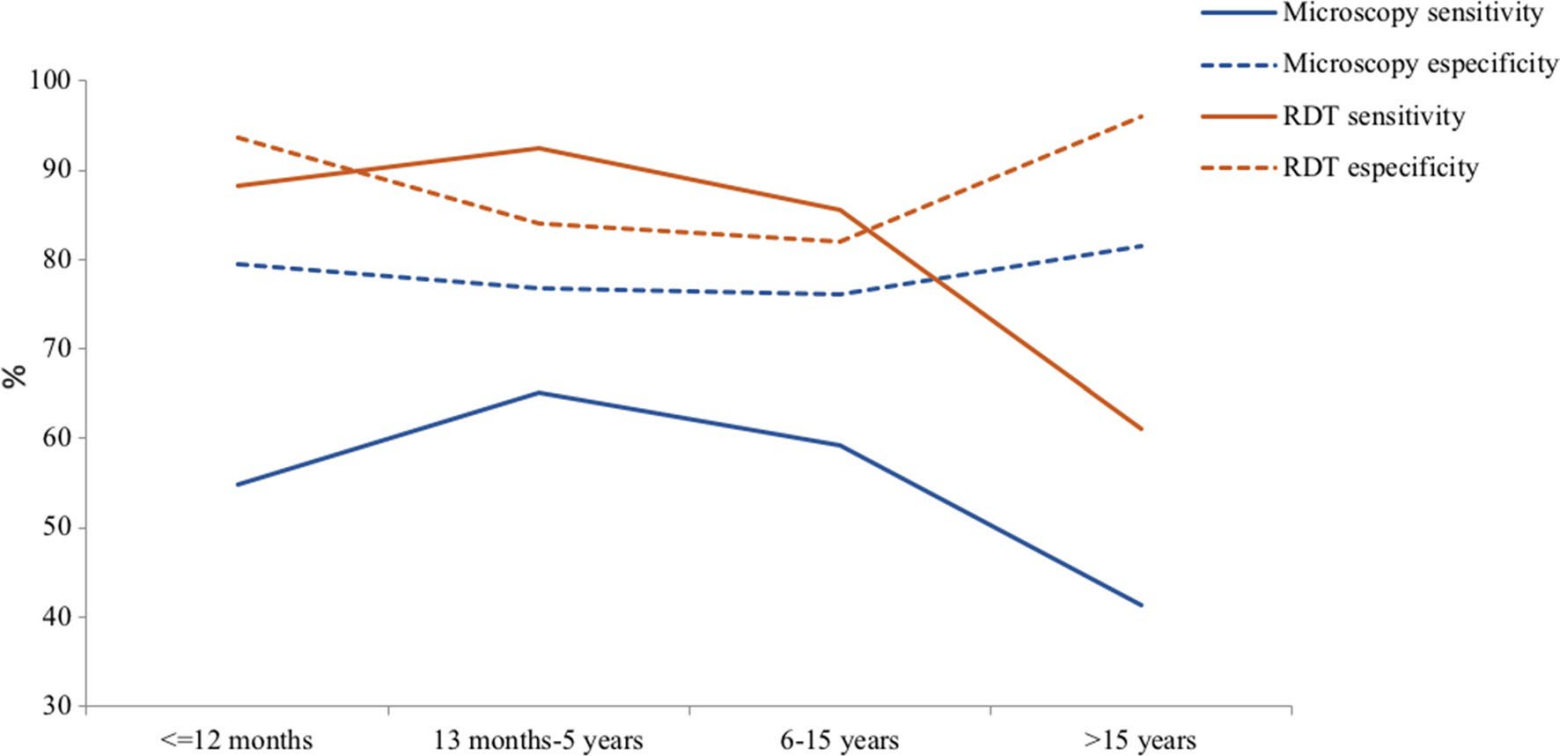
Graph of the sensitivity and specificity of microscopy and RDTs according to age. The graph shows how sensitivity decreases in both microscopy and RDTs; the older age the lower sensitivity of both techniques

RDT showed higher sensitivity than microscopy in all age groups. Both diagnostic tools showed higher sensitivity in children aged 13 months to 5 years, decreasing as age increase. RDTs also showed higher specificity than microscopy in all age groups, being both techniques more specific in children under 12 months of age and above 15 years old (Table 6, Fig. 4). The positive predictive value of the rapid test and microscopy increases with age between 2 months and 15 years. In the group of > 15 years it comes back down in the case of microscopy. On the contrary, the negative predictive value of the rapid test and microscopy decreases with age between 2 months and 15 years, increasing after 15 years (Table 6).

**Table 6:** Sensitivity and specificity between different groups of age

Age group	PCR		Total	SV (95% CI)	SpV (95% CI)	PPV (95% CI)	NPV (95% CI)
	Neg.	Posit.					
RDT vs. PCR							
≤ 12 months (n = 394)							
Neg.	282	11	293	88.17 (80.05–93.27)	93.69 (90.35–95.92)	81.19 (73.07–89.30)	96.25 (93.90–98.59)
Posit.	19	82	101				
13 months–5 years (n = 513)							
Neg.	243	17	260	92.41 (88.18–95.21)	84.08 (79.42–87.85)	81.82 (76.87–86.77)	93.46 (90.26–96.66)
Posit.	46	207	253				
6–15 years (n = 505)							
Neg.	137	49	186	85.5 (81.35–88.86)	82.04 (75.51–87.12)	90.60 (87.24–93.96)	73.66 (67.06–80.26)
Posit.	30	289	319				
> 15 years (n = 309)							
Neg.	171	51	222	61.07 (52.52–68.99)	96.07 (92.11–98.08)	91.95 (85.66–98.24)	77.03 (71.27–82.79)
Posit.	7	80	87				
Microscopy vs. PCR							
≤ 12 months (n = 394)							
Neg.	239	42	281	54.84 (44.73–64.56)	79.4 (74.48–83.59)	45.13 (35.52–54.75)	85.05 (80.71–89.40)
Posit.	62	51	113				
13 months–5 years (n = 513)							
Neg.	222	78	300	65.18 (58.73–71.11)	76.82 (71.62–81.31)	68.54 (62.07–75.02)	74 (68.87–79.13)
Posit.	67	146	213				
6–15 years (n = 505)							
Neg.	127	138	265	59.17 (53.86–64.28)	76.05 (69.04–81.89)	83.33 (78.41–88.26)	47.92 (41.72–54.13)
Posit.	40	200	240				
> 15 years (n = 309)							
Neg.	145	77	222	41.22 (33.16–49.78)	81.46 (75.11–86.48)	62.07 (51.30–72.84)	65.32 (58.83–71.80)
Posit.	33	54	87				

The table shows the sensitivity and specificity in different groups of age as well as the positive predictive value and negative predictive value. The most important thing is the decrease of the sensitivity of microscopy and RDTs with the age. *SV* sensitivity value, *SpV* specificity value, *PPV* positive predictive value (95% CI), *NPV* negative predictive value (95% CI)

## Discussion

Accurate diagnosis of *Plasmodium* species is important not only for establishing the correct treatment regimen, but also for applying effective malaria control strategies in endemic regions where the four species of malaria parasites exist, as in EG. Misidentification of the *Plasmodium* species could result in severe public health concerns due to inappropriate treatments, leading to recrudescence and even drug resistance [28]. Malaria control requires a high quality diagnostic method to detect the parasite before prescribing anti-malarial treatment following the WHO’s indications. Malaria parasitological diagnosis targets treatment, supports characterization of the treatment response, and enables early identification of the parasite [29].

Malaria is one of the main public health problems in EG and still a leading cause of morbidity, with more than 25% of its population infected with malaria parasites. *Plasmodium falciparum* contributes 90% of the malaria burden. This study highlights possible deficiencies of diagnosis by microscopy and RDT in Bata District, EG. SnM-PCR was used as the gold standard to compare the results obtained in EG by Giemsa microscopy and a RDT. A false negative rate of 19.4% and 7.4% was found for microscopy and the RDT, respectively. Alemu et al. [30] also used nested PCR as a reference technique in their study in the north of Gondar (Ethiopia), detecting a false negative rate of 13.1%. False negatives are a big public health problem because there is a part of the population that returns home without a correct diagnosis and treatment, not complying with the rule “fast and correct diagnosis, and treatment with confirmed presence of the parasite”. This could have important implications in health, transmission, and possibly mortality. Accurate diagnostic methods are the basis for an adequate disease control and avoiding resistance to antimalarial drugs or the spread of resistance. As diagnostic resources are limited in EG, without a reference laboratory, especially the Continental Region, microscopy remains the laboratory standard for diagnosing malaria. However, this study shows, once again, how molecular methods have generally been accepted to offer excellent sensitivity and specificity and are considered reference standards for the diagnosis of malaria infection [31].

According to the Malaria Microscopy Quality Assurance (QA) Manual from the WHO [32], it is necessary to ensure that healthcare professionals and patients have full confidence in the laboratory result, and the diagnostic results benefit the patient and community. Hospitals and health centres require expert microscopy for the management of malaria cases; it is the gold standard in endemic countries for identifying mixed infections, treatment failures, and quantifying parasite density. In the present study, microscopy showed less sensitivity (55.3%) and specificity (81.28%) than the RDT (83.74% and 89.11%, respectively) using SnM-PCR as the reference technique. Taking into account the age groups, it was observed a decrease with age of the sensitivity in microscopy as well as in the RDTs. Parasite density might have determined the positive infections detected by microscopy and RDTs [33], as the parasite density (from moderate to low) decrease with age [34]. This decrease might be also related to the immunity status. In malaria endemic countries, as EG, acquired immunity in adult is associated with the presence of submicroscopic infections that are more likely to be undetected by microscopy and RDTs [33, 35]. On the other hand, it was observed that RDT specificity values were higher when sensitivity declined. A study carried out by Laurent et al. found that the specificity of RDT varied with age and was inversely related to the prevalence of positive blood films in different age groups, that is, the specificity decreased as the prevalence of malaria increased. Moreover, malaria prevalence was higher in children under 5 years of age, when less specificity is detected [36]. Abeku et al. [37] also detected a decrease in specificity with decreasing age and increased prevalence of malaria in symptomatic patients. Another study reported a similarly low RDT specificity (52%) in symptomatic children younger than 5 years in an area of intense transmission [38]. The RDT decrease of sensitivity with age while specificity increases have been also described by Siahaan et al. According to this paper, both parameters were influenced by parasite density related to the improvement of their immune system (antiparasite disease) with age [39].

Regarding the differences found by setting, the sensitivity of RDT was greater in the rural area than in the urban area, while the specificity was lower in rural zones. In rural areas the transmission and endemicity of malaria is higher (people have greater immunity to malaria), allowing RDT to be more sensitive. It is known that the sensitivity of the RDT is affected by low parasitic densities, and that below 100 parasites/μl the RDT performance decrease [6, 36].

The specificity of RDTs in rural areas was lower than in urban areas, this may also be due to the fact that some patients maintain a high level of antibodies against malaria for more days or did not received adequate treatments [22].

The low parasite density along with the high number of false negatives detected in microscopy (19.4%), indicates that in many occasions it is difficult to give a good microscopic diagnosis. On the other hand, the microscopists in EG, at least the microscopists who participated in this study in the Continental Region, seem to need better training to accurately diagnose *Plasmodium* species. It was detected misdiagnosed due to erroneous readings performed by the laboratory technicians, bad staining of the slide, and stain artefacts, or wrong species diagnoses. Despite the inherent limitations of Giemsa microscopy for malaria, the quality of microscopic diagnosis largely depends on the quality of training. In a study carried out in Kenya, the diagnostic accuracy of malaria microscopy was positively associated with refresher training in microscopy. Therefore, the refresher training and QA programme should be systematically implemented together to improve parasitological diagnosis of malaria by microscopy [40].

Sometimes there may be individuals with submicroscopic infections, doing very difficult or impossible to give a positive diagnosis by microscopy or RDT. Submicroscopic infections have been reported in high transmission regions as in Ghana and hypo-endemic areas as in Uganda [41–43], demonstrating a relationship between submicroscopic infections and clinical malaria in children. These findings highlight the importance of treat patients with low-density malaria parasitaemia, and support interventions addressed to eliminate submicroscopic infections [44]. The concept that submicroscopic infection has clinical consequences strengthens arguments for malaria control strategies designed to eliminate all malaria parasitaemia. These strategies should include active search for asymptomatic patients to be treated with ACT, for their total cure [44]. In the near future, the authors want to study with the EG Malaria Programme the possible submicroscopic infections (asymptomatic, or chronic) and its effect in the transmission of the disease in EG.

Taking into account the samples with coinciding diagnosis in SnM-PCR, more diagnostic matches were found with RDT than microscopy. SnM-PCR allows for the detection of low-density infections and, even more importantly, mixed infections, which are routinely missed in microscopy, as this PCR has a somewhat lower limit of detection (approximately 0.0001 parasites/μl) [22], making it an ideal confirmatory test for malaria diagnosis.

In sub-Saharan Africa, HRP2 RDTs are the most commonly used test for parasitological confirmation of malaria before treatment, the test used for this study was based on the HRP2. Several reports have noted significant declines in the sensitivity of HRP2 RDTs after a decline in the intensity of transmission or deletion of *pfhrp2*. Parasites lacking *hrp2* are a potential source of false-negative HRP2 RDTs; the gene is absent in *P. falciparum* isolates with deletions in *hrp2* [45–48]. In this study, the false negatives in the RDT (128 samples). The 128 false negatives will be studied by PCR to determine whether they have a deletion in the *hrp2* gene or not.

Due to the sensitivity and specificity of the RDT, it is a good alternative for the diagnosis of malaria. Given the false negatives detected with the RDT, it is important to use the tests recommended by the WHO. The test could be selected from the “WHO list of prequalified in vitro diagnostic products” updated November 17, 2017. Occasionally, when *P. falciparum* parasitaemias are elevated, the LDH band could be positive, giving false positives of non-falciparum species. This could have implications in the epidemiological surveillance, but not in the treatment, since in Equatorial Guinea, all malaria infections are treated with ACT as a treatment of first intention.

Although RDTs are used as diagnostic methods, diagnosis by microscopy should never be abandoned because it is the gold standard in endemic areas. In addition, microscopy allows the calculation of parasitic densities and identification of all species and is cheaper than the other methods. Although it is the best diagnostic method with high sensitivity and specificity, PCR is still costly and not very useful for routine diagnosis.

## Conclusions

The choice of PCR method as the “gold standard” for comparison should have influenced the outcome of these findings as previous studies used blood film microscopy as their comparator. Even though the SnM-PCR is one of the best methods for malaria diagnosis, it still expensive and requires trained personnel and reagents that need to be frozen or refrigerated, which is sometimes difficult in the country, but the technique is good for epidemiological studies or to test the efficacy of control methods applied in the area. In this study, a high number of false negatives were detected by diagnosis with RDTs. Therefore, an exhaustive study of the deletion of the HRP2 gene must be done in EG to help choose the correct RDT for this area. In addition, this study provides information about the necessity of training in microscopy. A network of reference centers could potentially support ongoing diagnostic and control efforts by malaria control programs in the long term. The National Centre of Tropical Medicine (Madrid, Spain) currently supports the National Programme against Malaria of Equatorial Guinea to perform all of the molecular studies necessary for disease control, such as SnM-PCR for the diagnosis of malaria and nested RFLP-PCR for the study of mutations related to resistance in *P. falciparum*.

This study shows a variation in the performance of the RDT with respect to the study area and age. These sociodemographic characteristics must be taken into account, in order to explain the results obtained. This, in a certain way, indicates that the use of RDTs is limited, it may be useful for diagnoses in remote areas, but for prevalence studies it is better to continue using microscopy as a reference technique or if it is possible the SnM-PCR.
